# The effect of spatial boundaries on memory in a virtual environment

**DOI:** 10.3758/s13421-026-01852-y

**Published:** 2026-01-26

**Authors:** Julie C. Lamont, David K. Bilkey

**Affiliations:** Department of Psychology, University of Otago, 275 Leith Walk, Dunedin, New Zealand

**Keywords:** Event boundary, Spatial navigation, Doorway effect, Virtual reality, Memory

## Abstract

Crossing a spatial boundary, such as a doorway, often signals the ending of one episode and the beginning of another, segmenting ongoing experience into events. When conducted in real environments, this ‘doorway effect’ differentially affects memory for objects encountered within and between events. The evidence for this occurring in immersive virtual reality (VR) environments is mixed. The present study investigated the ‘doorway effect’ in a VR environment and also examined how event memory is affected when segmentation processes are disrupted by interference tasks. Ninety participants explored a five-room VR building containing interactable objects. During exploration, some participants were presented with virtual distractor tasks that required either visuospatial working memory or simple rapid-reaction responses. These tasks occurred either at doorways or in the middle of rooms. Later recall for the temporal order and contextual location of objects was examined and compared with controls, who explored the building without any distractions. The results showed that a doorway effect was evident in the control, no-distractor condition. The visuospatial distractor task impaired memory for objects, but only when it occurred in the middle of a room, possibly because it separated the representation of the encapsulating room into different events. When this task was performed in the doorway, however, its effects overlapped with, and did not add to, the spatial boundary effect. Together these findings show that both spatial boundaries and spatial distractor tasks can segment memory for experience in an immersive VR environment.

## Introduction

Despite the continuous nature of conscious experience, the world is understood as a complex series of discrete events that have beginnings and endings, and that tend to be perceived consistently. For example, Newtson and Engquist (1976) found that participants showed remarkable agreement when explicitly asked to mark breakpoints when watching short films of human activities. In other studies, participants have been shown to generate consistent boundary placements, both intrasubjectively and intersubjectively, even when retested after a year delay (Speer et al., 2003). Furthermore, the processes underlying the differentiation of event boundaries have recently been identified at a neurophysiological level (Baldassano et al., 2017; Ben-Yakov & Henson, 2018; Ezzyat & Davachi, 2021).

Event segmentation is now recognised as a fundamental cognitive process with implications for basic functions such as perception and memory. For example, individual ability to infer events from ongoing activity, known as ‘event segmentation’, is directly related to later memory for the activity (Sargent et al., 2013) and the ability to perform everyday actions (Bailey et al., 2013). Event Segmentation Theory (EST; Zacks et al., 2007) describes how the segmentation of activity into events interacts with memory in terms of the creation of mental representations of situations, known as ‘event models’. A ‘working event model’ specifically refers to an individual’s representation of the activity that is currently unfolding in real time. When this representation is held in working memory for the duration of the event it can be used to inform predictions as to what will happen next. When salient features of the situation change dramatically, however, the disparity between predictions and outcomes grows (Radvansky & Zacks, 2017). When this occurs, EST proposes that the current event model is updated to better match the evolving situation, while the increased processing results in better long-term memory for the event (Zacks et al., 2007).

Given the importance of spatial boundaries in the mapping and segmentation of physical space (Burgess et al., 2001; Peer & Epstein, 2021), it is perhaps not surprising that crossing these boundaries can trigger the segmentation of events (Brunec et al., 2018; Lee, 2017). Previous research has shown that cognitive processing is disrupted when spatial boundaries, such as doorways, are crossed. For example, participants are more error-prone and are slower to respond when probed about out-of-sight objects they are carrying, after they have moved through a doorway to another room, compared with when they have walked the same distance within a single room (Radvansky & Copeland, 2006). The event horizon model proposes that the doorway is an event boundary cue, segmenting the environment representation into two distinct event models (Radvansky & Zacks, 2014). Termed the ‘doorway effect’ or the ‘location updating effect’, the memory deficit for items in another room is thought to demonstrate difficulty in accessing information from the previous (and therefore less relevant) event model, because it is no longer in working memory (Pettijohn & Radvansky, 2016). This doorway effect is also evident in the structure of long-term event memory (Horner et al., 2016; van Helvoort et al., 2020), indicating that details from different rooms are encoded as different events because of the spatial boundary between the rooms. In contrast, details within a room are more closely bound together in a unified mental representation and are therefore stored together, and more easily retrieved together (Zacks et al., 2007).

In summary, a body of research indicates that the demarcation of the physical world by spatial boundaries provides anchor points for the demarcation of events in event memory. This doorway effect has been described not only in real environments but also in partially immersive environments, where participants navigate through an environment presented on monitors (Radvansky et al., 2011). When the doorway effect has been tested in fully immersive VR environments, however, the results have been variable. McFadyen et al. (2021) reported that moving through a doorway in VR had little effect on memory for objects. Logie and Donaldson (2021) found that doorways had little additional effect on memory for word lists over and above that provided by temporal segmentation. In contrast, however, van Helvoort et al. (2020) found a doorway effect for image recognition in an immersive VR task, as did Li et al (2025), although in the latter case for conceptual rather than spatial boundaries. Each of these studies varied in terms of such task features as contextual cues in the room and the opportunity to interact with the environment. The aim of the current study was to first confirm that a doorway effect could be generated in an immersive virtual environment for memory for object order and object/context relationships where interaction with objects and room contextual cues were provided. We also explored how a distracter task, that occurs either in doorways or within a room, affects memory for objects that have been encountered previously. Two versions of the distractor task will be tested, each placing greater or lesser demands on spatial memory. We predict that the distractor task that more fully engages spatial memory, will have a greater decremental impact on object/place memory, because it will interfere with the spatial memory processing that occurs in regions such as the hippocampus as spatial boundaries are traversed (Burgess et al., 2002). The distractor intervention will occur either at the room boundary or within the room, and we hypothesise that it will have a greater impact on long-term memory for objects when conducted at doorways by disrupting processes by which information relevant to the ‘just-leaving’ environment that is currently stored in working memory is transferred to, or updated in, long-term memory to create the unified memory representation of that room. To achieve this, we created a virtual reality exploration task that required participants to engage with objects throughout a series of rooms whilst being interrupted by these distractor tasks. These distractor tasks were imposed either at spatial boundaries (at doorways between each room) or between them (at the centre of each room), A control condition was also run whereby the exploration task was completed with no intervening distractor tasks. Participants were tested on their long-term memory for the temporal order of the objects and for the context (room) that they were found in, because previous studies have indicated that these are hippocampal-dependent operations (Fortin et al, 2002; Maurer & Nadel, 2021).

## Methods

### Participants

Ninety-nine participants were recruited through the University of Otago’s research participation scheme or via word of mouth. Participation initially operated on a voluntary basis, but reimbursement in the form of undergraduate course credit or NZ$20 was later implemented to aid recruitment. All participants were required to be at least 18 years old. The data from five participants were excluded due to technical difficulties as a result of distractor tasks failing to trigger. One participant was excluded for misinterpreting the test questions, and one participant was excluded because they had the shortest exploration time (the time spent in the virtual building/encoding environment, excluding the duration of any distractor tasks) and performed below chance on all memory performance variables, indicating that they had made no genuine attempt at the task. Two participants were also excluded for being outliers with abnormally long exploration times. The final sample therefore consisted of 90 participants (40 men, 48 women, two nonbinary) aged between 18 and 63 years (*M* = 22.9, *SD* = 7.1), with the majority (91%) 30 years or under. All participants had normal or corrected-to-normal vision as based on self-report. Corrective glasses were worn by 32/90 participants during the procedure. Several participants (4/90) also self-reported red-green colour-blindness. For both these subgroups, their mean ‘doorway effect’ data was well within 1 standard deviation of the overall mean and trended in the direction of the whole dataset. Self-report of VR experience revealed that 92% had minimal previous experience, the remainder ‘moderate’. Ethical approval was gained by the University of Otago Human Ethics Committee.

### Materials

The experiment used a desktop PC, with the VR environment displayed on an HTC Vive headset and an HTC Vive wireless controller used for participant interaction. Within the environment, a gloved virtual hand model was visible to simulate the participant’s right hand via controller tracking. Google SketchUp (http://www.sketchup.com/) and Unity (https://unity3d.com/) were used to create the VR architecture.

### Memory encoding virtual environment

**Fig. 1:**
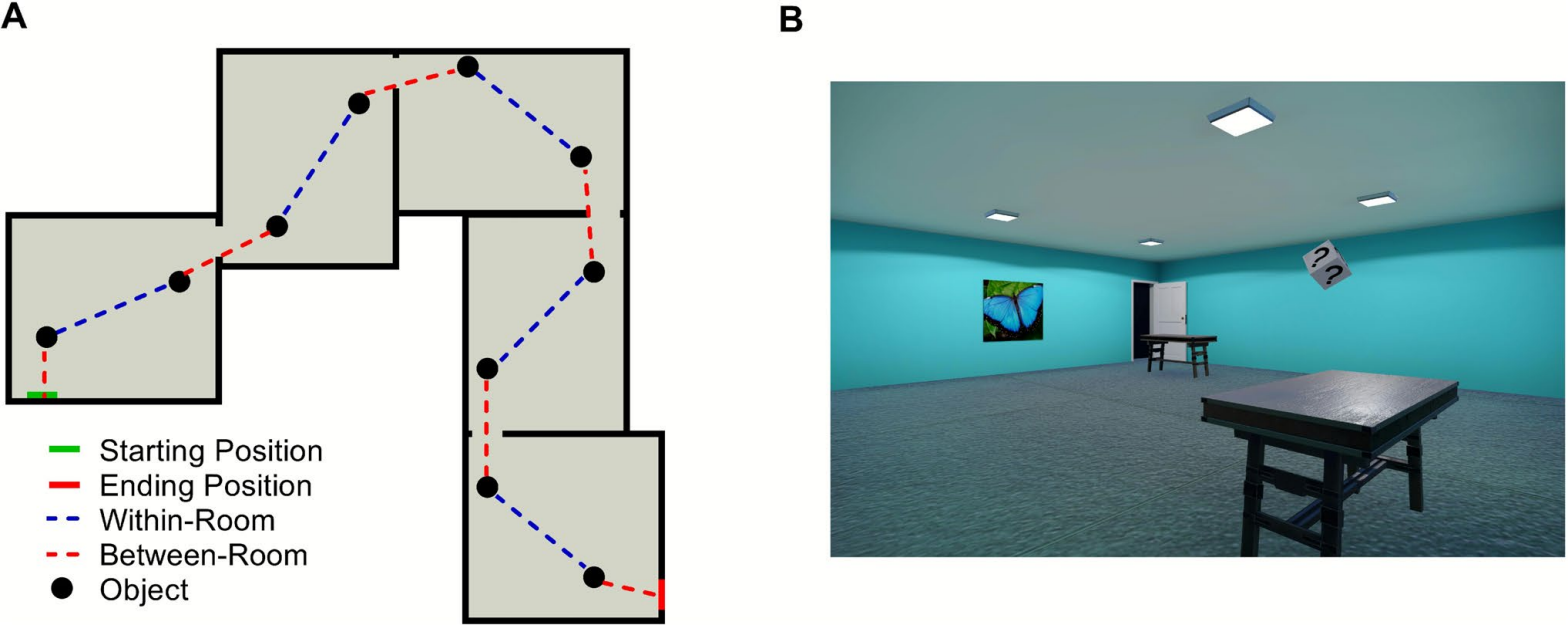
Virtual reality building. **A** Plan view schematic of the virtual building, illustrating the spatial relationships between rooms and objects. **B** A first-person perspective of a room. Two tables, wall art, a doorway into the next room, and an object’s placeholder cube are visible. (Colour figure online)

The virtual building environment was comprised of five rooms in a single-storey layout (see Fig. 1). Each room was connected to two other rooms (excluding the very first and last rooms), creating a single route that the participant had to follow, thereby reducing variability in the amount of information encoding and consolidation opportunities. Pressing a button on the handheld trackpad moved the participant 60 cm (virtual distance) forwards within the environment, in the direction the controller was pointing when the button was pressed. Movement and related visual updates occurred in discrete steps rather than continuously, to decrease the possibility of cybersickness (Daşdemir 2023). User data was logged every 200ms from gameplay, specifically local *x-y* coordinates, *x-y-z* head rotations, and the period that a participant ‘held’ an object.

Adjoining rooms were connected through doorways with open doors. The floor and ceiling remained the same throughout the building. To convey changes in location, the five rooms were distinguishable from one another by their shape (all 72 m^2^) and wall colour. As an additional identifier, given that some individuals may struggle with colour discrimination, each room had a unique photo of an animal on the wall opposite its entrance doorway that roughly matched the colour of the room (e.g., a red parrot in the room with red walls).

Inside each room were two empty rectangular tables, all placed an equal distance of 6.5 m from the previous and the next table. Tables served as locations for the target objects that the participants were tasked with familiarising themselves with. The 3D objects that were generated on top of the tables were modified from Unity’s Asset Store, selected as being a common everyday item that could be handheld. Some example objects were a torch, drink bottle, and a soccer ball. There were two objects in each room, which resulted in ten objects in total. Each object was located on the same table for each participant. Model objects included unique materials/textures and behaved consistent with the laws of earths physics and gravity. Participants in the control condition moved freely along the route between rooms and memory for object order and context were tested in a within-subjects design. Participants in other conditions were presented with distractor tasks either in the middle of rooms or at doorways.

### Virtual distractor tasks

Participants in these conditions were randomly allocated to one of four groups that encountered distractor tasks of two different types at two different locations during their virtual exploration. At the point of distractor task initiation, participants were virtually transported into a large, open virtual environment with an interactive panel of blocks on a wall straight ahead. The wall panel housed nine 9-cm square blocks in an irregular arrangement, each protruding 2.5 cm from the panel. In this environment only, the participant’s tracked virtual hand was constrained to a fist with the index finger extended in a pointing position. The tasks were performed while standing stationary and the ability to virtually step was deactivated.

Some participants were exposed to a visuospatial sequencing distractor task which was a modified version of the Corsi block tapping task (CBT) for visuospatial working memory (Brunetti et al., 2014; Corsi, 1972; Nelson et al., 2000). The CBT is conceptually similar to the well-known digit-span test, where the participant stores a number sequence of increasing length in working memory (Kessels et al., 2008). For each trial, blocks lit up individually in a random sequence, flashing blue for 500 ms with an 800-ms break between each sequence member. The final block of the sequence lit up green instead of blue, indicating to the participant that the full sequence had been presented. The participant was then prompted to reproduce the given sequence from memory by virtually tapping on the blocks in the same order, with the blocks lighting up upon touch. The generation of each random sequence involved random sampling with replacement, meaning an individual block could appear multiple times in a sequence. If the participant reproduced the sequence correctly, indicated by the tapped blocks lighting up blue/green, then the sequence length increased by one block in the next trial. If an incorrect cube was tapped, indicated by the blocks lighting up in red, the trial stopped, and a new sequence of the same length began. Previous fMRI data show that the performance of the CBT is associated with activation in the hippocampus (Toepper et al., 2010).

Other participants were exposed to a simple reaction distractor task that operated in the same fashion as the visuospatial sequencing task but with a fixed sequence length of only one block per trial. For each trial, a singular random cube lit up blue for 500 ms. The participant was then instructed to tap the lit cube as quickly as possible, with the cube lighting up green upon touch if correct or red if incorrect. After an 800-ms break, the next trial began with a new random cube. Instructions emphasised accuracy in addition to speed, to encourage engagement of attention. The CBT and the reaction task both involved similar movements, perceptual and attentional processing—however, the CBT required the use of spatial memory, whereas the reaction task did not. Both distractor tasks were run for a set duration with a visible timer counting down for 25 s, and prior instructions that emphasised both accuracy and speed. The experimenter monitored VR performance throughout the procedure to ensure participants were on-task.

### Postexploration questionnaire

The postexploration questionnaire asked forced-response multi-choice questions about event details from the virtual building exploration. Specifically, recall for the temporal order and environmental context was tested for each of the ten objects. Each object had two questions concerning its chronological position within the sequence of all objects: ‘Which object came immediately after the object above?’ and ‘Which object came immediately before the object above?’. Therefore, for each object one question referred to a pairing with the other object in the same room (within-room relationship), and the second to a pairing with an object in an adjacent room (between-room relationship). The environmental context question for each object asked: ‘Which room was the object above in?’. For all thirty questions, an image of the target object was displayed at the top centre of the screen and images of the other nine possible objects (in the case of temporal order questions), or of the five rooms (in the case of context questions) were displayed below the target object in a random order. An additional ‘None’ option was also displayed for temporal order questions, being the correct option for the questions referring to the between-room relationship for the very first and very last objects. The participant was required to click on the radio button next to the image of their chosen answer below the target object. Participants were instructed to take their best guess if they did not know the answer.

### Pretrial training

Prior to the experimental trial, participants completed a VR tutorial to familiarise them with the user basics of VR, the experiment’s primary navigation/memory task, and with the appropriate virtual distractor task (if any). Participants were explicitly informed that their sequential memory and context memory would be probed. A practice postexploration questionnaire was administered after the VR tutorial environment. This was complete with model answers and replicated the format of the ultimate retrieval task. Due to space confinements of the physical real-world room, participants essentially stood stationary and used a controller with their right hand to navigate through the environment. Participants were able to take a step or two within the 2.4-m × 2.1-m playing area, visibly outlined within the virtual world if the controller was detected to be close to the perimeter. The controller had two active buttons: the trackpad button on the front, clicked with the thumb, moved the player forward virtually 60 cm in the direction it was pointing; and the trigger button on the back, squeezed by the index finger, engaged with interactable objects if virtually within 5 cm of proximity.

Once the headset was fitted and the tutorial environment was generated, a virtual diagram hint labelled ‘Step’ accompanied text instructions until the trackpad was clicked, to assist with learning the basic movement controls. A practice post-exploration questionnaire was also administered using details from the VR tutorial environment, complete with model answers, to ensure that all task requirements were understood before proceeding.

### Study phase

The experiment’s study phase required virtual exploration through the five-room building, which had ten interactable objects to be studied. Participants started in a large open environment containing a wall and a black-filled doorway that connects to the first experiment room. The player’s view initiates facing the wall with text instructions adjacent to the door. After stating that the trial begins once they step through the doorway, instructions reiterate that the focus of the trial is to remember as much detail as possible about the building’s layout and contents. Instructions also explain that there is no time limit to explore, that it will not be possible to return into a previous room once it has been exited, and that they may encounter ‘challenges’ (the distractor tasks) along the way to be completed before they can proceed. The participant begins by using the trackpad to step through the door, which behaves as a teleportation portal into the first experiment room.

In each experiment room, participants were required to locate the visible ‘placeholder cube’—a small white cube with a question mark on each of the six faces—positioned at eye level above a table (see Fig. 1B). When a participant approached the placeholder and reached out their virtual hand to touch it, it became outlined in yellow, signalling to the participant to squeeze the trigger button on their controller to interact with it. Upon this interaction, the object to be encoded is generated in its place, giving a 10-s opportunity for familiarisation before disappearing. All participants were exposed to the same objects in the same order (see Appendix). Participants had the choice to engage with the objects by virtually picking them up or to instead observe them from a distance. At the point of object generation, the next placeholder cube simultaneously appears. Such a system of placeholders was introduced to prevent two objects being viewed more closely together if in the same room rather than across adjacent rooms (Horner et al., 2016). Once the second object was generated, the third placeholder (located in the second room) becomes visible, and so forth for all ten objects across the five rooms.

Participants moved through the rooms freely, and for those in the control condition the object relationship comparison (within room or between room) was tested within-subject. The progress of participants in the other conditions was interrupted by one of the virtual distractor tasks either as they crossed through each doorway, or as they crossed through the centre of each room. This part of the study was run with a mixed design with the task location (doorway vs. room centre) and the task type (spatial sequence vs. reaction task distractor) comparison being between subject, while the object relationship comparison (within room or between room) was within subject. Once teleported to the distractor task environment, a visible ‘3-2-1 GO’ timer begins immediately to prepare the participant for the start of the task. Both the visuospatial sequence and simple reaction tasks ran for 25 s before reaching completion. Distractor tasks were encountered five times during exploration, and each instance of the distractor tasks was independent from performance on prior instances (the visuospatial task always began with a sequence length of four blocks to be remembered). Upon completion of the distractor task, the participant was instantly transported back to the main experiment room, just in front of their last position; either a step in front of the centre of the room or a step in front of the entrance doorway.

Exploration continued until all five rooms had been explored. Participants were unable to cross into the next room until both objects in the current room were activated, providing the same order of objects for everyone. Similarly, participants could not return to the previous room once it had been explored. The experimental procedure finished once the last doorway, connecting the fifth room to an empty black space, is crossed by the participant. This final event teleported the participant from the experiment environment to a user interface, that contained text explaining that the virtual exploration had ended and to remove their VR headset. Those in conditions where distractor tasks were encountered in doorways completed their task for the fifth time at this final doorway before reaching the trial-end user interface.

### Test phase

The experiment’s test phase was administered on a computer using the web-based survey platform Qualtrics (https://www.qualtrics.com/). A filler task was first implemented to prevent working memory maintenance—mental repetition of just-learned material to retain it in short-term memory—which would have typically led to superior long-term memory performance (Hartshorne & Tal Makovski, 2019). For this filler task, a random starting number between 100 and 500 was presented on the computer screen, and participants mentally counted backwards in multiples of threes for 1 min before entering their resulting number (Brown, 1958; Peterson & Peterson, 1959). After entering their result, the survey progressed onto the post-exploration questionnaire.

The 30 forced-response questions were presented on screen one by one, with no time limit in place. For each object, the two temporal order questions were asked in a random order, followed by the object context question. This question ordering was to ensure that the images of the rooms did not cue the retrieval of contextual information that could aid in recall for the temporal order of objects. After completing an object’s three questions, the participant moved on to the next object’s questions, the order of objects being randomised. Once the full questionnaire was completed, participants were debriefed and thanked, with the opportunity to give anonymous feedback or ask any questions before receiving any reimbursement and leaving. Experiment sessions took approximately 30–40 min in total.

### Data analysis

For each temporal-order and object-context question, responses were classified as either correct or incorrect. Memory ability was expressed by both temporal order recall, scored as the percentage of correct responses to the twenty temporal order questions, and by context recall, as the percentage of correct responses to the ten object context questions. To examine the effect of spatial boundaries on memory, each participant’s temporal order recall was separated by the spatial relationship between objects to form two further measures: recall for pairs of within-room objects, calculated from questions whereby where the object in question (cue object) and the correct answer (target object) belong to the same virtual room; and recall for pairs of between-room objects, whereby cue and target objects belong to adjacent rooms separated by a doorway.

Using the logged exploration behaviour data, the amount of time spent between the generation of each object and its successor was calculated for both the first objects in each room and the second objects in each room. This provided measures of within-room time (time spent between the two within-room objects) and between-room time (time spend between the last object in one room and the first object in the next). Each measure therefore includes the encoding period spent familiarising oneself with the object (up to 10 s), and the postencoding period before the next object is reached, excluding the duration of distractor tasks if encountered. Each time total was used to calculate the percentage of the overall exploration time spent within rooms and between rooms. However, if the interaction with an object’s placeholder cube was shorter than 250 ms, it was possible for the generation to go undetected—as was the case for 3% of objects across participants. The time also could not be measured for the last object, for it had no successor object to be detected. To bypass these shortcomings, the calculations for within-room and between-room time percentages were based on the mean amount of time between the generation of detected objects. Thus, the sum of the two proportions did not necessarily equal 100% for each participant.

A single time-factor variable was created to represent how disproportionate each participant’s exploration time was for the different areas of the virtual building, calculated as (total time spent between rooms minus total time spent within rooms)/(total time spent between rooms plus total time spent within rooms). A Doorway Effect score was also calculated for each participant as their arcsine-transformed temporal order recall for within-room objects minus arcsine-transformed recall for between-room objects. Correlation analyses were performed by group to investigate how the time factor related to the magnitude of the doorway effect and how the doorway effect correlated with two proxies of engagement in the environment: mean duration of interaction with an object and mean rotation of view (azimuth) per second.

A series of analyses were performed on the measures of memory ability and logged exploration data. Within-subjects factors were analysed for the control group, with paired-samples *t* tests. For all other participants between-subjects factors were analysed with either between-subjects or mixed analysis of variance (ANOVA) or covariance (ANCOVA). ANCOVA was used instead of ANOVA whenever appropriate to account for the effect of exploration duration. Significant ANOVA/ANCOVA effects were followed up with simple main effects analyses in the form of one-way ANOVA/ANCOVAs. Dunnett’s comparisons were used to compare the performance of controls with the performance of each distractor task group. When the data from percentage scores violated a parametric test’s assumption of normality, scores underwent an arcsine transformation to reduce the skew. A log10 transformation was additionally applied if the data did not contain values of zero. If transformed data still violated a test’s assumption of normality, a nonparametric test was used on the raw data instead—specifically, the Wilcoxon signed-rank test instead of paired-samples *t* test. Or, when no such alternative is available, the intended parametric test was used despite the violation, with the understanding that ANOVA *F* tests are robust to such violation of normality (Blanca et al., 2017; Schmider et al., 2010) Measures of effect size are reported alongside each analysis: *r* = Z/√n for Wilcoxon signed-rank test; Cohen’s *d* for paired-samples t tests and Dunnett’s comparisons; and generalised eta-squared for ANOVAs and ANCOVAs (Bakeman, 2005). For tests on transformed data, descriptive statistics as expressed in terms of the raw data while test statistics refer to the transformed data.

## Results

### Doorway effect for temporal order

To determine whether there was an overall effect when data from all conditions were combined, a Doorway Effect score was calculated for each participant as their arcsine-transformed temporal order recall for within-room objects minus arcsine-transformed recall for between-room objects. Therefore, a positive Doorway Effect score indicates a memory advantage for within-room objects compared with between-room objects, and a negative score a memory disadvantage for within-room objects (an anti-doorway effect). The mean Doorway Effect score was 4.8 ± 2.0 which was significantly greater than zero, *t*(89) = 2.34, *p* =.022. We also tested whether the overall Doorway Effect was associated with level of engagement with the task, as approximated by measures of object engagement and view rotation. Neither of these measures was significantly correlated with the Doorway Effect (both *r* <.1, *p* >.2)

**Fig. 2:**
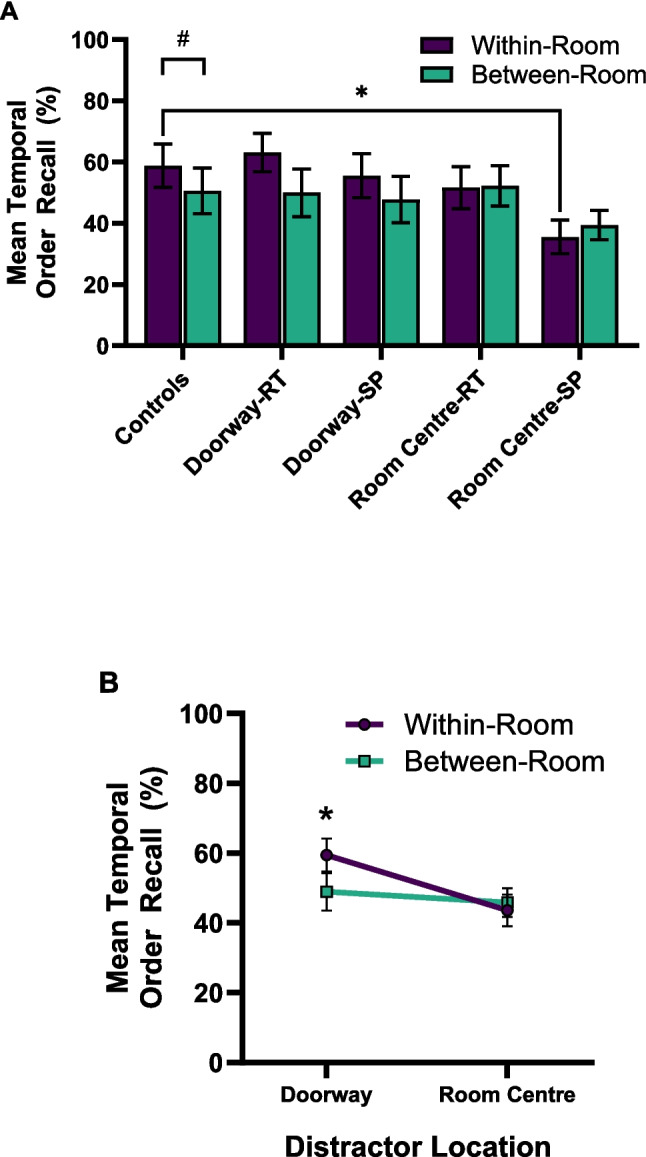
Temporal order recall by distractor task group. Analyses were performed on arcsine-transformed data. Error bars = ±*SEM*. **A** Mean temporal order recall for pairs of within-room and between-room objects (# = significant Wilcoxon signed rank test, *p* =.05. * = significant Dunnett’s comparison, *p* ≤.05). **B** Recall for temporal order of objects. There was a significant two-way ANCOVA interaction for the factors of all distractor task locations and object relationship (*p* ≤.05) and a significant Dunnett’s comparison (*p =.05) task location effect in the doorway group. RT = reaction task distractor, SP = spatial distractor. (Colour figure online)

Participants’ recall for the temporal order of neighbouring objects was compared for within-room and between-room pairs of objects in the no-distractor, control group (Fig. 2A, left bars). A Wilcoxon signed-rank test using the *z*-score approximations and continuity correction indicates that the control groups’ recall was greater for pairs of within-room objects than for between-room objects (*Z* = 1.96, *p* =.050, *r* =.65), with a mean difference of 8.2% (*SD* = 16.7%). Thus, a ‘doorway effect’ was evident in recall of temporal order after uninterrupted exploration of the virtual environment.

To assess whether the distractor tasks differentially disrupted recall, a 2 (task location; doorway vs. room centre) × 2 (task type; spatial sequence vs. reaction task distractor) × 2 (object relationship; within room or between room) mixed ANCOVA for arcsine-transformed temporal order recall, that controlled for exploration duration, was performed for distractor-task participants (i.e., Fig. 2 excluding controls). The results revealed a significant main effect of object relationship (within the same room vs in different rooms), *F*(1, 68) = 5.31, *p* =.024, η^2^_G_ =.01. There was also a significant interaction between object relationship and task location, *F*(1, 68) = 8.83, *p* =.004, η^2^_G_ =.01 (Fig. 2B). The main effects of task location and task type were not significant, *F*s ≤ 2.34, *p*s ≥.131, η^2^_G_s ≤.01, nor were any other interactions, *F*s ≤ 1.09, *p*s ≥.301, η^2^_G_s ≤.01. Object relationship was analysed for each task location, controlling for exploration duration, using one-way ANCOVAs. The results revealed (Fig. 2B) that the within-room/between-room difference was statistically significant for participants who encountered distractor tasks at the doorways, *F*(1, 35) = 13.9, *p* =.001, η^2^_G_ =.03, but not for those who encountered them at the centre of rooms *F*(1, 34) = 0.25, *p* =.621, η^2^_G_ <.01. For the doorway-distractor groups, temporal order recall was therefore greater for within-room objects (*EMM* = 59.3%, *SE =* 4.6%) than for between-room objects (*EMM* = 48.8%, *SE* = 4.6%), while the room centre distractor groups showed no significant difference between within- and between-room recall (*EMM* = 43.8%, *SE =* 4.7%, and *EMM* = 46.0%, *SE =* 4.7%, respectively).

The arcsine-transformed, within-room object recall, versus between-room object recall of controls was then compared with each of the four distractor-task groups (Fig. 2A). The results of Dunnett’s comparisons showed that temporal order recall for pairs of within-room objects is significantly worse for interrupted exploration compared with uninterrupted exploration only when the interruption was from the visuospatial sequence task conducted in the middle of rooms (Room Centre-SP), mean difference = −32%, *p* =.050. Between-room recall did not differ from that of controls for any of the distractor task groups (*p*s ≥.559). Together these results show that encountering the visuospatial task in the centre of rooms impairs subsequent recall for the temporal order of objects within that room, thereby eliminating the doorway effect, within-room, memory advantage.

### Object context recall

**Fig. 3:**
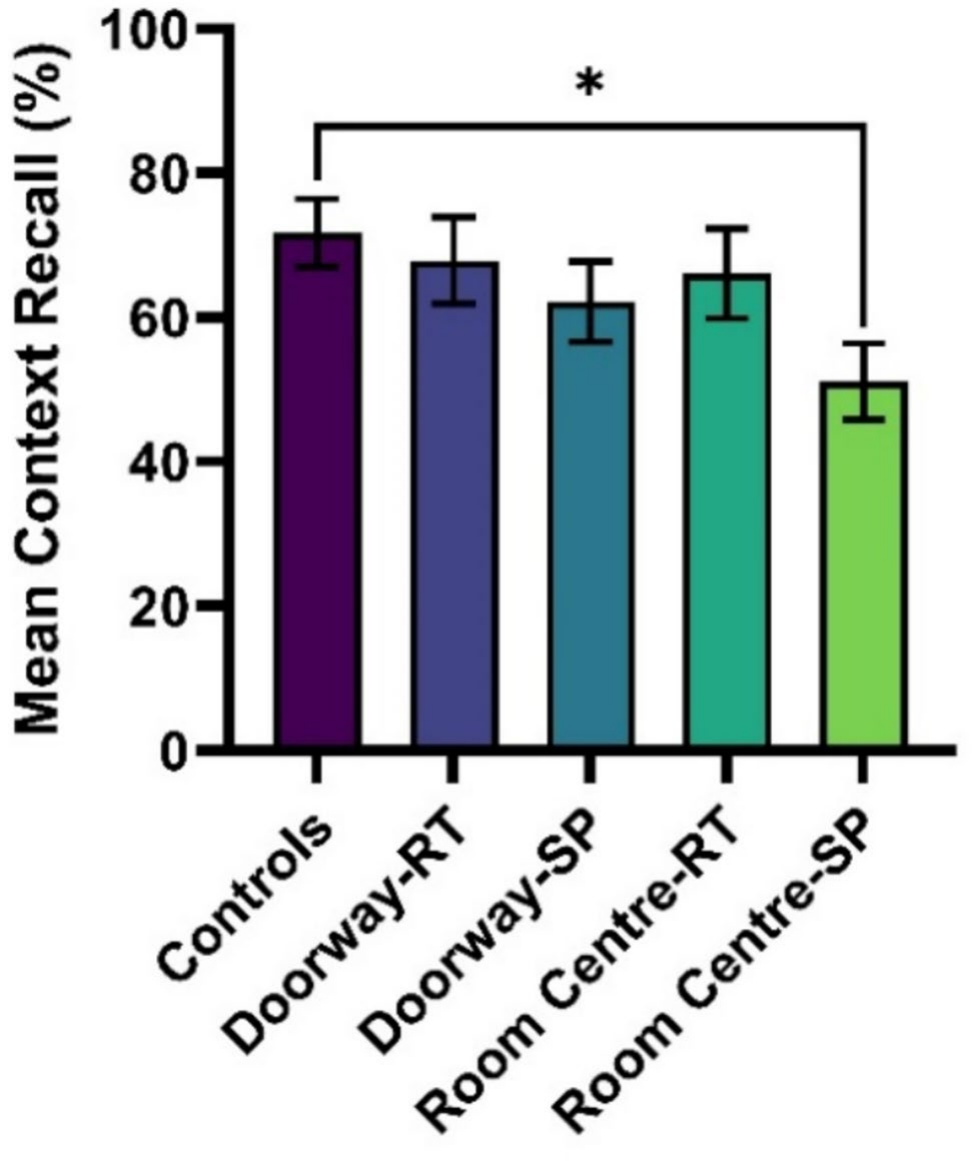
Mean object context recall by distractor task group. Analyses were performed on transformed data. Error bars = *SEM*; * = significant Dunnett’s comparison (*p* ≤.05). (Colour figure online)

Raw data for the room that each object was presented in (context recall) is visualised in Fig. 3. The arcsine- and log10-transformed context recall data was analysed with a 2 × 2 between-subjects ANCOVA, with the factors of task location (doorway vs. room centre) × task type (spatial sequence vs. reaction task), and with exploration duration as a covariate. The ANCOVA, which excluded the control data, failed to detect any significant main effects or interactions, *F*s ≤ 2.92, *p*s ≥.092, η^2^_G_s ≤.04, indicating that the type and location of distractor tasks does not differentially affect ability to recall objects’ locations (context) from the exploration. To test for differences between the control data and the other condisions, we ran corrected Dunnett’s comparisons between the control group transformed context recall data and each of the four distractor task conditions. The results indicate that object context recall for an interrupted exploration is significantly worse than for an uninterrupted exploration only if the interruption was from the visuospatial sequence task conducted in the centre of rooms (mean difference = −20%, *p* =.045).

### Time between objects

Measures of within-room time and between-room time consist of any time spent encoding an object or post-encoding time before the next object, calculated for the first and second objects from each room respectively. The control groups proportion of exploration time spent between-rooms (*M* = 59.4%, *SE* = 7.6%) was significantly longer than that spent within-rooms (*M* = 43.2%, *SE* = 5.4%), *t*(16) = 5.33, *p* <.001, *d* = 1.29. Therefore, controls spent more time during the encoding and/or post-encoding periods for the second objects in each room, which involved crossing through a doorway, compared with for the first objects in each room, which required traveling the same virtual distance without crossing a doorway.

Further analyses were conducted to assess whether the participants in groups that performed distractor tasks also displayed similar temporal patterns. One participant was removed from these analyses due to software failing to detect the generation of more than half of the objects. To investigate whether people that had a relatively short or relatively long total exploration duration differed in how they distributed their exploration time between objects, participants were categorised into quartile-based exploration duration bins, specifically representing the 25% of participants with the shortest exploration duration (25%), the lower-mid 25% (50%), upper-mid 25% (75%), and the top 25% with the longest exploration duration (100%). A 2 × 2 × 2 × 4 mixed ANOVA with the factors of distractor task location (doorway vs. room centre) and type (spatial sequence vs. reaction task), exploration location (within-room vs. between-room), and exploration duration quartile (25% vs. 50% vs. 75% vs. 100%), was performed for the proportion of exploration time. The results revealed a significant main effect of exploration location, *F*(1, 56) = 60.29, *p* <.001, η^2^_G_ =.50. This main effect was modified by a significant interaction with distractor task location, *F*(1, 56) = 15.00, *p* <.001, η^2^_G_ =.20. Main effects for task location, task type, and exploration duration quartile were not significant, *F*s ≤ 3.37, *p*s ≥.072, η^2^_G_s ≤.01, nor were any other interactions, *F*s ≤ 3.20, *p*s ≥.059, η^2^_G_s ≤.06. One-way ANOVAs were used to analyse the simple main effect of exploration location for each distractor task location. The results revealed that the effect of exploration location was statistically significant for both the participants who encountered distractor tasks at the doorways, *F*(1, 36) = 58.0, *p* <.001, η^2^_G_ =.59, and for those who instead encountered them at the centre of rooms *F*(1, 35) = 9.79, *p* <.001, η^2^_G_ =.21. The doorway-distractor groups tended to spend a greater proportion of their exploration time between-rooms (*M* = 60.0%, *SE =* 1.4%) than for within-rooms (*M* = 41.8%, *SE* = 1.1%), as too did the room centre-distractor groups (*M* = 53.4%, *SE =* 0.1% and *M* = 47.7%, *SE =* 0.1%, respectively). This effect occurred despite between-room and within-room objects being an equivalent spatial distance apart. In other words, participants tended to spend more of their exploration time during the encoding/post-encoding periods of objects if the period included crossing into a new room, though to a lesser extent for those who encountered distractor tasks in the middle of rooms.

### Temporal distances and memory performance

We then considered how distributions of exploration time within the building related to subsequent recall for the objects. It is possible that long-term memory for the temporal order of objects is better for objects that are encoded more closely together in time. If this were the case, then the extent to which more time was spent between-rooms compared with within-rooms should directly correspond to the extent that temporal order recall was better for within-room objects compared with between-room objects (magnitude of the Doorway Effect). A single time-factor variable was created to represent how disproportionate each participant’s exploration time was for the different areas of the virtual building, calculated as (total time spent between-rooms minus total time spent within rooms)/(total time spent between rooms plus total time spent within rooms). The distance between within-room and between-room objects was approximately equal, and therefore, a time factor of 0, which indicates an equal amount of time spent between-room and within-room, suggests equivalent time spent exploring, or near, the objects. A time factor >0 indicates a between-room dwell preference, and <0 a within-room preference. Correlation analyses were performed by group to investigate how the time factor related to the magnitude of the doorway effect. The results showed no significant relationship between the two variables for any of the groups, *r*s ≤ ±.31, *p*s ≥.220. The observed doorway effect is therefore independent of how much time is spent for within-room object pairs relative to between-room object pairs.

## Discussion

In this study, we investigated memory for objects and contexts in participants exploring an immersive virtual building. We were interested in whether a doorway effect could be detected, and how encountering distractor tasks at doorways impacted temporal order and context memory. First, we found that a doorway effect was evident when participants were allowed to explore the virtual environment uninterrupted. That is, control participants recall for the temporal order of objects was better when the pair of objects in question were contained within the same room, rather than spanning across adjacent rooms (separated by a doorway). This occurred despite the objects all being the same physical distance apart, and the effect was independent of temporal distances between objects. This result is consistent with that of van Helvoort et al. (2020), who found a doorway effect for images in VR, and extends this effect to memory for temporal order of virtual objects. It is unclear how much the degree of immersion in the VR environment is a factor, as previous studies have reported a doorway effect in minimally immersive environments (Radvansky et al., 2011). Furthermore, our proxy measures of immersion (object manipulation time and view rotation) were not correlated with the overall doorway effect. Even though our study used a more enveloping simulation than that used in previous studies, there is a lack of cues such as the vestibular and proprioceptive information about self-movement that would accompany a journey through a real environment. This suggests that it may be conceptual nature of the transition between rooms that is more important than the cues that describe the physical environment (Li et al, 2025).

Although the absence vs. presence of a spatial boundary was not the only difference between pairs of within-room objects and between-room objects (pairs of within-room objects shared the same background contextual information, specifically the colour of the room’s walls and matching animal photo) this context was not provided as a cue during retrieval (Godden & Baddeley, 1975). Furthermore, when using a screen-based procedure similar to the present experiment, Horner et al. (2016) found a spatial boundary effect for temporal recall in long-term memory even if the two halves of each room had different wallpaper, as did van Helvoort et al. (2020) in a 360° immersive virtual museum environment with only one wall colour, thus ruling out changes in background contextual information as a complete explanation of the doorway effect.

When we examined the effect of a doorway-located distractor task on memory for temporal order, our data showed that this intervention, whether spatial or nonspatial, had little impact on the doorway effect, with memory for between-room items still being poorer than for within-room items. This result did not support our hypothesis that it would disrupt the consolidation of memory for the current event model (the objects in the room that was being exited from). One possible explanation is that the distractor task itself generated an event boundary (Wang & Egner, 2022), which masked any effect generated by the doorway itself. In contrast, however, if a spatial distractor task was undertaken in the centre of the room it had a negative impact on temporal order recall for within-room objects. These data support the idea that the spatial distractor task created an event boundary. What is interesting, however, is that this effect did not occur for the reaction task distractor. This suggests that it was the spatial nature of the task that is important. Previous research shows that the hippocampal region of the brain encodes episodic memories by binding together disparate event elements from various neocortical regions into a unified ‘event engram’ (Horner et al., 2015). This brain region is also involved in processing spatial memory and context (Lisman et al., 2017), likely as a part of the ‘where’ of episodic memory. Furthermore, engagement with the Corsi block-tapping task, on which our spatial distractor was based, is tied to hippocampal activity (Toepper et al., 2010). It is possible, therefore, that the spatial component of the distractor task, was particularly effective in disrupting hippocampal processing while object information was being integrated with within-room contextual cues. Furthermore, when distractor tasks occurred in the middle of rooms, and were therefore misaligned with spatial boundaries, the experience of exploring a room was fragmented. An inability to incorporate a distractor task occurring in the middle of a room into the room’s event representation might result in the formation of twice as many event representations (Radvansky & Zacks, 2014)—one for the first half of the room before the distractor task, and one for the second half of the room afterwards. This would not, however, explain the greater impact of the spatial distractor task, although one cannot rule out the possibility that it was the difficulty of the task that might have been important. Further research would require that distractor tasks are matched on this dimension.

Our data were consistent with those by DuBrow and Davachi (2013), who found that when participants studied images of faces and objects, memory was relatively enhanced for image order when the images in question belonged to the same context. This memory enhancement was eliminated when a difficult arithmetic task was added between the presentation of each image during encoding along with an emphasis on processing each item more deeply. These data were interpreted as showing that the associative binding between images was disrupted by the additional task, impairing storage and subsequent recall. In short, DuBrow and Davachi (2013) found that adding cognitive load between all stimuli eliminated a context-based boundary effect that was otherwise present. In our study, cognitive load was selectively added either at spatial boundaries or between spatial boundaries, and it was only when cognitive load was added between boundaries that the doorway effect was eliminated. Thus, boundaries impair the ability to recall sequential relationships between items directly interrupted by those boundaries, regardless of whether they are in the form of contextual boundaries, spatial boundaries, or task switching/cognitive load.

Our procedure allowed participants to freely roam through the building (though they were restricted from returning to a prior room). We unexpectedly found that the location at which participants encountered distractor tasks affected where in the building participants spent their time while exploring. Excluding any time performing the distractor tasks, those participants who had uninterrupted exploration without distractor tasks, and those who encountered distractor tasks in doorways, had a relatively longer encoding/post-encoding period for objects when the period included crossing into a new room, compared with periods that included walking to the next object in the same room. Those who encountered distractor tasks in the middle of rooms did so to a much lesser extent, spending closer to an equal proportion of time between objects regardless of whether there was a spatial boundary between them or not. From the perspective of EST, it is likely that event boundaries served as natural breakpoints, with participants dwelling at boundaries to recapitulate the experienced event (Zacks et al., 2007). Our results suggest that spatial boundaries were stronger dictators of dwelling preferences compared with boundaries from distractor tasks, given that groups who encountered distractor tasks in the middle of rooms still tended to spend more time between rooms compared with within rooms. This may be because the physical demarcations provided by the doorways directly related to the overarching goal to remember information from the virtual environment. That is, the doorways divided up the building into separate rooms, and this spatial information formed the scaffolding for a cognitive map of the segmented environment (Brunec et al., 2018; Peer & Epstein, 2021; Robin, 2018). On the other hand, the distractor tasks only cue event boundaries to the extent that they are a sudden change in perceptual information and a temporary shift to a subordinate goal. The importance of individual boundaries is therefore determined in part by where they fall on the event hierarchy, with perceived higher-level events taking priority over lower-level events.

As regards context memory, participants had poorer memory for the room in which each object was in if they had endured additional virtual distractor tasks throughout their exploration, but only if such distractor tasks required visuospatial working memory and were encountered in the middle of rooms. This effect is possibly due to the different types of cognitive engagement that occurs in response to the different distractor task demands. If memory impairments because of distractor tasks were a simple matter of additional event boundaries, similar impairments to context memory would be expected for both tasks, as the simple reaction task provides the same change in perceptual information and a similar goal switch as the visuospatial task—the CBT—both likely initiating the formation of an event boundary. EST postulates that ongoing perception involves the maintenance of an event model of the current situation in working memory (Zacks et al., 2007). Encountering a distractor task in the middle of a room directly interrupts the event model for that room. Given that processing for CBT performance, and processing for context, likely overlap, it is possible that the spatial task takes cognitive resources away from event cognition (Griffiths & Fuentemilla, 2020). This would directly affect the encoding of event model information such as the objects’ spatial context. Our results suggest, however, that the event model can still be maintained in working memory so long as the task does not also use visuospatial working memory, hence why the reaction task did not produce a deficit in memory for context. If the critical process is event model maintenance, then this would explain why neither distractor task affected event memory when they occurred at spatial boundaries. That is, whether visuospatial working memory is engaged or not during a distractor task is irrelevant to event memory if it does not interrupt a working event model.

In conclusion, we provided more evidence to show that a doorway effect can be generated in an immersive virtual reality environment. Furthermore, we have shown that the doorway effect can be extended to memory for temporal order of objects. We also showed that engaging in extraneous distractor tasks during encoding impaired later recall if the distractor required visuospatial working memory and occurred in the middle of rooms. The same task produced no significant effects if it occurred at doorways, nor did a similar task that occurred in the middle of rooms but did not require visuospatial working memory. Finally, we found that participants typically spent more time during and/or immediately after an object’s encoding period if that period required entering a new room. This could reflect an instinctive memory strategy that encourages participants to recapitulate prior information at physical boundaries.

## Figures and Tables

**Fig. 4:**
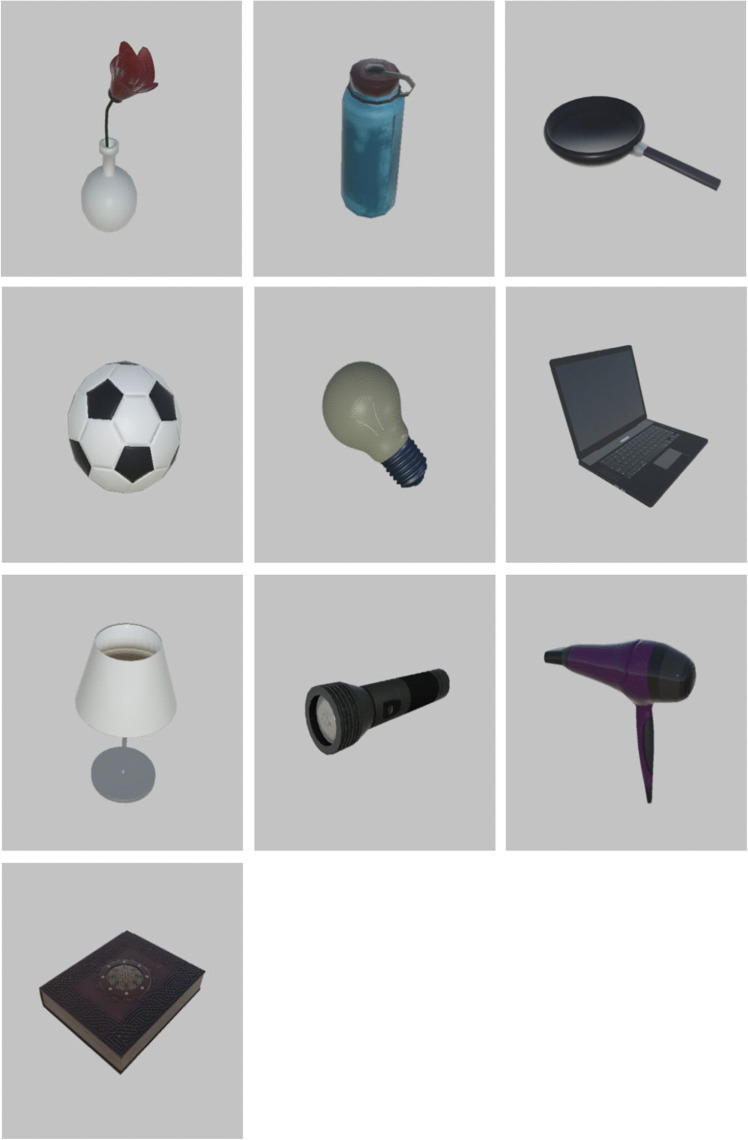
Objects presented in the study phase of the experiment

## Data Availability

Anonymised data is available from the authors on request

## Appendix

Fig. 4

## Code availability

Code is available from the authors on request
